# Sex Assignment in West Africa: A Challenge for a Pediatric Surgeon—Culture Matters

**DOI:** 10.4269/ajtmh.20-0207

**Published:** 2020-08

**Authors:** Luis F. Rivilla

**Affiliations:** Division of Pediatric Surgery and Urology, Ramon y Cajal University Hospital, Madrid Spain; Department of Biomedical Sciences, San Rafael Nebrija Center, Antonio de Nebrija University, Madrid, Spain

Aminata was a 10-month-old patient who was born with ambiguous genitalia, consisting of severe clitoral hypertrophy and an imperforated vagina. The morphology of the genitals showed clear masculinization. She had grown up healthy with breastfeeding and had caused her father intense joy. He was happy that his first child from his marriage was a boy and named this son Johannes. Johannes’s parents were humble farmers from a rural area in Northeast Liberia and brought him to see us because he had small inguinal masses on both sides since birth. They were concerned about bilateral inguinal hernias. When we initially saw Johannes, he was alone with his mother because his father delayed his trip for reasons related to the livestock they owned. The mother was tired after wandering a considerable distance from their village for 2 days.

Our initial evaluation revealed that Johannes had ambiguous genitalia. Abdominal ultrasound showed a uterus and ectopic ovaries along with a salpinx in the inguinal area. We explained that to surgically correct the ambiguous genitalia, and the inguinal masses, would require feminizing genitoplasty. In Monrovia, it was impossible to carry out cytogenetic and hormonal studies as we would have performed in our European hospital.

Initially, we decided to wait for the father. However, the next day, the mother agreed to perform the operation, sooner rather than later, because the father would delay his arrival for several more days for reasons related to the crops. The following day, we decided to operate under general anesthesia, and a reductive clitoriplasty, bilateral herniorrhaphy with reduction of the ovaries to the abdomen and a vaginoplasty with vulvoplasty were performed. The immediate postoperative period was uneventful. The girl returned to breastfeeding smoothly within 24 hours, and her mother seemed happy from the beginning, assigning her daughter the new name of Aminata.

For more than 10 years we have worked together with surgeons and local health workers to develop pediatric surgery missions providing surgical treatment and postoperative care of children with congenital malformations from the neonatal period to adolescence, mainly in the digestive, urogenital, and craniofacial areas. These experiences have been developed in hospitals from the Catholic Hospitaller Order of Brothers of Saint John of God, in various countries of West Africa, such as Senegal, Sierra Leone, Liberia, Togo, Benin, and Cameroon, where there was traditionally a teaching and healthcare link with our hospitals or universities in Europe.

Every year, we have learned to commit fewer mistakes and to be more efficient in our objectives with children who are born with severe malformations. We frequently try setting goals and ideals similar to those we use in our Western hospitals. Sometimes, we forgot to adapt to the social or cultural demands of children and their families. Frequently, families have worries other than the strictly medical problems we see.

On the fourth postoperative day, the father arrived from the village and everyone celebrated his reunion with joy. That night, when the nurse went to cleanse the surgical wounds, the father saw his child’s female genitalia and was completely surprised. The next morning during rounds, we found the mother crying and unable to nourish her baby properly. The nurse informed us that at night they had heard the couple arguing, as well as some screaming. The father had rushed out that same morning. When we went to review the surgical scars, we were astonished to recognize a deep burn that covered the entire front of the trunk from the neck to the genitals and perineum. When we asked the mother about the burn, she answered in a whisper in her local Mandingo language. She said that the father poured boiling water on the baby at night, to reverse the evil changes the white doctors had done to Johannes. The father blamed the mother for everything that happened to their son.

Promptly, we transferred the girl to the theater to perform a wound cleaning with sedation. Subsequently, we spoke with the mother so that she could ratify her statement to the local police in Monrovia and search for the person responsible for this shocking aggression. Jointly with the hospital management, we would register an official complaint, of which we never knew if he was discovered and tried for these events.

During the following days, we performed the therapeutic protocol for childhood burns, with a satisfactory result after 2 months. We maintained contact with the local surgeons, who reported that she was growing naturally. The female morphology of her genitalia was satisfactory at 6 postoperative months with mild sequelae of the burns.

As health workers and especially surgeons, we think merely of pathology, its technical approach, and the resolution of postoperative care. However, occasionally, there are frightening consequences of forgetting to address the situations of the family in which our small patient lives. Diseases are influenced not only by education of the general environment but also by the secondary implications from traditional and ancestral beliefs, based on religious, ethical, or historical motivations.

Genitourinary malformations are one of the most common, and those that affect their morphology, such as the diverse forms of male or female genital dimorphism, experience a significant impact not only on the patient himself but also on the family and the sociocultural environment in which they live, being rejected in western society and also in Africa. Those children can be abandoned since birth as we have evidenced in some of the aforementioned countries.

We have made efforts to avoid inappropriate selection of patients. Still, performing genital surgery on children with ambiguous genitalia collides with many diverse beliefs and cultural traditions in West Africa. However, these facts can make us reflect on the short time we invest in the West to take into account the cultural or moral aspects of the malformation with which our patient was born and how important they can become.

When we have subsequently developed other surgical missions, we have not forgotten Aminata or her mother, as well as all those people with ancestral traditions or rigid moral postulates.

Focused education of healthcare personnel, public awareness programs, and improvement of diagnostic facilities through international collaboration like short-term missions in global pediatric surgery, may improve the outcome and minimize psychological morbidity of those most fragile. Our duty to preserve the health and well-being of our patients must be above beliefs and traditions; however, we must avoid judgments and learn to be sensitive to alternative management approaches, knowing that culture matters.

